# Locating Hotspots for the Social Life Cycle Assessment of Bio-Based Products from Short Rotation Coppice

**DOI:** 10.1007/s12155-021-10261-9

**Published:** 2021-03-25

**Authors:** Daniela Fürtner, Lea Ranacher, E. Alejandro Perdomo Echenique, Peter Schwarzbauer, Franziska Hesser

**Affiliations:** 1 Wood K plus–Competence Center for Wood Composites and Wood Chemistry, Kompetenzzentrum Holz GmbH, Altenberger Straße 69, 4040 Linz, Austria; 2 Department of Economics and Social Sciences, Institute of Marketing and Innovation, University of Natural Resources and Life Sciences Vienna, Gregor-Mendel-Straße 33, 1180 Vienna, Austria

**Keywords:** Social life cycle assessment, SLCA bio-based products, Socioeconomic impacts, Socioeconomic indicators, Indicator selection, Stakeholder analysis

## Abstract

The establishment of new value chains raises expectations in economic and social benefits. To determine whether these expectations can be fulfilled or whether there are also negative consequences, social aspects should be assessed as early as the R&D phase. Potential social impacts can be assessed with the help of a social life cycle assessment (SLCA). A common problem in SLCA studies is the large number of social aspects. Thus, it is important to prioritize the most relevant aspects. Scholars agree that socioeconomic indicators should not be selected on a purely intuitive and common sense basis and that a standardized approach is missing. A three-step process has been developed to identify the most vulnerable and relevant social aspects. These three steps were implemented into a case study to empirically test the method. Short-rotation-coppice as an alternative form of agricultural dendromass production is one possibility to obtain wood resources for the processing of bio-based products. The use of agricultural land for dendromass production promises additional income for the region’s farmers and job opportunities for the local population. The extant literature shows that the most frequently addressed impacts are related to workers’ health and safety aspects. The outcome of this study aims to support future research by identifying an appropriate approach for the selection of indicators in SLCA. For studies with a similar focus, the proposed set of indicators can be used as a framework in itself or serve as a basis for the choice of relevant social indicators.

**Supplementary Information:**

The online version contains supplementary material available at 10.1007/s12155-021-10261-9.

## Introduction

The emerging bioeconomy is seen as one of the major drivers in climate change mitigation and for the development of a more sustainable future. In the context of this paper, the bioeconomy is understood as defined by the European Union [1] in their bioeconomy strategy—covering “all sectors and systems that rely on biological resources (animals, plants, microorganisms, and derived biomass, including organic waste), their functions, and principles (including all primary production sectors that use and produce biological resources (agriculture, forestry, fisheries, and aquaculture).” Cost-efficiency and sustainability of bio-based value chains are both strongly linked to regional feedstock availability. Spatial proximity of feedstock production, as provided by Short Rotation Coppice (SRC) dendromass production, may have positive impacts on transportation costs and the environment [2]. Ensuring sustainable development of evolving value chains requires an accompanying sustainability assessment, which provides information about (potential) sustainability implications. For a holistic view on sustainability, a life cycle approach has to comprise all three fields of sustainability: environment, economic, and social [3]. An established technique for sustainability assessment of longstanding development is life cycle assessment (LCA), as one of the most frequently used methods [4].

New value chains, as aimed for in the bioeconomy (“knowledge-based bioeconomy”), are a young field of research, and knowledge about the social dimension of the related production processes is limited. The increasing interest in bio-based products requires a precise monitoring of entailing the social or socioeconomic impacts. Information on important social aspects allows decision- and policy-makers to improve the social performance and prevent undesirable social implications. Regarding bio-based value chains, a high social risk potential is assumed for the upstream processes in the agricultural sector [5]. However, comparison of social impacts is especially challenging for bio-based value chains, since socioeconomic effects are strongly dependent on several production factors like the cultivation method, land quality, and the production scale [6]. In addition, through the relation to the spatial context, the effects have a strong regional dependency, since socioeconomic impacts have an influence on the respective environment of production locations [7]. In the case of bio-based products from SRC, two points are striking: studies regarding the production method SRC as well as regarding the geographical scope of interest, Eastern Europe or specifically Slovakia, are missing.

A method suitable for this purpose is social life cycle assessment (SLCA), analogous to conventional LCA for the assessment of environmental implications. SLCA is an ISO-compliant technique for the assessment of (potential) social impacts with the aim to assess “social and socioeconomic aspects of products [and services] and their potential positive and negative impacts along their life cycle encompassing” all stages of a products’ life cycle from “cradle to grave,” including raw material extraction, manufacturing, distribution, use, re-use, maintenance, as well as recycling and final disposal as stated by Benoît et al. [8]. The underlying goal of SLCA is to foster the area of protection (AoP), which is usually human dignity and well-being [9]. Human well-being is defined by social, human, physical, financial, and natural capital, which contribute to the present and future well-being of individuals [9]. However, SLCA is, in contrast to LCA, a very young field of research and still under development. Although LCA and SLCA differ in many aspects, the development of the SLCA method seeks to follow the same structure as proposed for LCA in ISO 14040 and ISO 14044, comprising goal and scope definition, inventory analysis, impact assessment, and interpretation of the results [8].

Due to many years of focusing on environmental and economic aspects, social impacts are still under-investigated, which also concerns the social performance in the production of bio-based products [5]. So far, information about social aspects that are relevant to be considered in SLCA is lacking. As a consequence of the large number of socioeconomic impacts that may arise, the impact categories must be adapted to the individual case. Besides data collection, a research design for a socioeconomic assessment must be created with emphasis on expressive and comprehensive indicators, which is a challenging [10] but crucial and vitally important part in SLCA [11]. There is no standardized method for the selection of indicators in SLCA. Therefore, several experience-based approaches are used to make a selection. Indicators need to be individually adapted, since socioeconomic effects vary strongly across projects or cases, depending on different factors [12]. Especially the implementation of bioeconomy strategies requires a regional context-specific approach [13], which determines social aspects that are of core interest for potentially affected stakeholders.

Benoît et al. [8] provide an overview of social aspects related to five different stakeholder groups that should be addressed in an SLCA in the “Guidelines for SLCA of products” (referred to as UNEP/SETAC Guidelines in the remaining study). In literature, the terms social issues, effects, impact categories, subcategories, and indicators are used but cannot always be clearly defined as their conceptualization is quite diverse. Falcone et al. [14] described indicators as a bridge linking subcategories and impact categories. A standardization of the terms, as already stated by Kühnen and Hahn [15] as well as by Martin et al. [16], would be necessary for easier and more efficient orientation in the jungle of social aspects. A range of terms frequently used in relation to SLCA indicators was identified. Table 1 gives a range of definitions of these terms in order to enable a deeper understanding of them.

**Table 1:** Definition of terms used in SLCA

Term and hierarchy	Definitions
Social aspects TOPICS	Describes the subject to be measured [17]; also defined as social performance [18]; anything related to human well-being; any number of general topics (objectives, social issues, opportunities, indicators, indices, impact categories) [19]—description of the general topic.
Social effects EFFECTS	A social phenomenon’s causes induced by changes; social effects can cause impacts [20]—social effects are related to social aspects
Social impacts IMPACT	Describes consequences, caused by changes, influencing peoples’ lives directly [20]; “are everything that affect people” stated by Vanclay et al. [21]—therefore, impacts are caused by effects
Social (impact) ategory STRUCTURE IN SLCA	Describes a broad area of influence; each category is defined by a number of aspects [17]; referred to as social index in Siebert et al. [19]; every index characterized by one or several indicators; describes unknown cause-effect-relationships [19]—used for a structured description of the impacts
Social (impact) subcategory SUB-STRUCTURE IN SLCA	Describes a more refined classification of the term category; subcategories are understood as a compilation and combination of social topics, issues, aspects, and also effects, which are used to be measured and validated by one or several indicators. The UNEP/SETAC Guidelines [8] define the term subcategory as “social and socioeconomic issues of concerns”—used as a sub-structure of categories
Social indicator MEASUREMENT IN SLCA	No overall accepted definition [22]; a sign, symptom, or signal that shows something; gives information if something is existing or true [22, 23]; measures an aspect, one aspect can be measured by several indicators [17]—used to measure impact (sub-)categories in SLCA

For simplicity, the term social aspect is used as an overall description of anything related to human well-being, based on the explanation by Siebert et al. [19]. Furthermore, the terms social (impact) category and subcategory will not be divided, as a precise distinction between terms cannot be guaranteed. However, an exception would be the use of citations and discussion of literature, to not misrepresent statements of other authors. In this manner, the terms social and socioeconomic will be conflated, as their differences are not always obvious. For a more fluent reading experience, the terms aspects, impact categories and indicators are prefaced by social only.

All these terms can address negative and positive impacts. Regarding a sufficient SLCA, positive impacts should be assessed that go beyond compliance stipulated by laws [8]. Positive social impacts are often underrepresented in SLCA studies [24], but have recently received more attention, for example, from Ekener et al. [25] or Benoît-Norris et al. [26]. Positive social impacts are also known as social handprints, as proposed by Norris [27]. The major difference between LCA and SLCA lies in the possibility to also assess positive impacts in SLCA, which are always directly related to people who are (potentially) affected by these impacts. Thus, one of the most important questions is, which groups of people are (potentially) affected. This question serves to form stakeholder groups that are addressed in the SLCA. Relationships between effects and stakeholders may extend and overlap, since some effects can have impacts on several stakeholder groups simultaneously. Especially impacts on workers or local communities are strongly linked to the (national) society too (e.g., occupational disease affects a worker’s well-being and, at the same time, the health system and, in turn, society will also be affected to some extent). One possibility is to differentiate between direct and indirect effects. Identifying the stakeholder groups concerned needs to be a priority, but as is the case for indicator selection, there are no clear standards for identifying stakeholders.

The choice of indicators can restrict the social topics addressed in an SLCA study. The crucial step of indicator selection thus already defines the results that can be drawn from the study. It is essential to pay close attention, to set the focus on those social aspects, that are of particular relevance for the stakeholders concerned. There is a vast number of prevalent indicators but a lack of generalized and standardized indicators reflecting social performance clearly [15]. Due to the high numbers of indicators used in SLCA, the indicator selection process becomes a bottleneck, not the availability of indicators [28]. In order to address those social aspects that are particularly critical and relevant, consulting stakeholders and experts consultation is of high priority [29–32], as it is recommended by the UNEP/SETAC Guidelines [8]. A review of SLCA studies by Martin et al. [16] shows, that stakeholder input is used in most of the studies to consider the relevant social indicators. Multi-criteria-decision approaches can be used to weight selected indicators via experts’ scores (using a Likert scaling) [20]. This approach has been broadly utilized for the past 15 years [33]. Attention must be paid to what kind of stakeholders are engaged in the study, because perceptions regarding the importance of impacts and indicators will vary since such perceptions are subjective [16]. Stakeholders directly affected can give a thorough insight into their risks and needs but representatives should reflect the risks and needs of a larger number of individuals or of entire stakeholder groups. Additionally, public decision-makers are of core interest, since they can actively influence the effects through regulatory measures [29]. It is valuable to pay close attention to the indicator selection process to ensure efficient data collection, which will result in an effective assessment [34]. The cause and effect chains in SLCA are not as obvious as in conventional LCA [19], which makes the choice of indicators even more complex. In addition to stakeholder-specific social aspects, SLCA has to consider regional and context-specific social aspects [19] as well. To choose relevant social aspects on a global, regional, national, and sub-national level was also suggested by Bracco et al. [35]. This challenges the standardization of indicators, which takes a high priority in the development of the method [15, 36] and makes it necessary to develop indicators on sectoral and regional levels.

The aim of this paper is to provide a guiding framework for the identification of social and socioeconomic aspects and indicators relevant for the SLCA of bio-based value chains, produced from SRC dendromass, especially in Eastern Europe. To tackle the under-investigated social dimension, we developed a framework built upon the work of Siebert et al. [19] and applied it to a case study demonstrating its feasibility. Thereby, we propose a set of social aspects and corresponding indicators to be used in SLCA studies. This should help to save time and resources in later studies on the methodological choice on the one hand and through the availability of a final set of indicators for comparable studies on the other hand. Therefore, three different steps for the selection of social impact categories and indicators are proposed and tested. These steps including a literature review, stakeholder consultation, and social risk mapping with a specialized tool, to answer the following research questions:

- Which SLCA guidelines and sustainability standards are relevant for bio-based value chains from Short Rotation Coppice dendromass?
- Which social aspects and indicators are relevant for the socioeconomic assessment of bio-based products from Short Rotation Coppice dendromass?
- Which social aspects and indicators are prioritized by stakeholders to be included into the SLCA of bio-based products from Slovakian Short Rotation Coppice dendromass?

## Materials and Methods

Our study is built upon three different steps for the identification of relevant social aspects and indicators in SLCA. These three steps are applied and tested on a case study as described in the following.

### Description of the Case Study

The case study carried out investigations of the social aspects of relevance, caused by SRC-based dendromass production in Eastern Slovakia. A new value chain is being established in a demonstration project with the aim of regional dendromass production in Slovakia, feeding into the cascading use for several bio-based materials. For this purpose, fast-growing poplars are cultivated in Short Rotation Coppice (SRC) on marginal agricultural land, harvested in short intervals with a high level of mechanization. From a legal point of view, the planting of trees on agricultural land in Slovakia is regulated by Act no. 220/2004 Coll., on the Protection and Use of Agricultural Land (“Soil Protection Act”) [37]. Land that is classified with the quality from 5 to 9 in the code of eco-land evaluation unit (“ELEU”) or contaminated land can be cultivated with SRC for a maximum of 20 years [37]. Poplars from SRC as a source for dendromass are more widely established in the field of energy production but also represent one way to gain a natural resource in the immediate vicinity for processing industries.

The Slovakian Ministry of Agriculture and Rural Development has been leading the development of a national bioeconomy strategy since 2018. To date, Slovakia is already part of the Central-Eastern European Initiative BIOEAST, an initiative for knowledge-based agriculture, aquaculture, and forestry in the bioeconomy, to foster a sustainable bioeconomy [38]. Employment and turnover are considered as a proxy for the socioeconomic relevance of the bioeconomy sector. In Slovakia, 174,000 people were employed in the bioeconomy sector in the year 2015 [39]. The turnover of the Slovak bioeconomy in 2015 was at 11 Billion €, with the second highest share in the country being attributed to the agricultural sector [39].

### Proposed Methodology for Indicator Selection

**Fig. 1:**
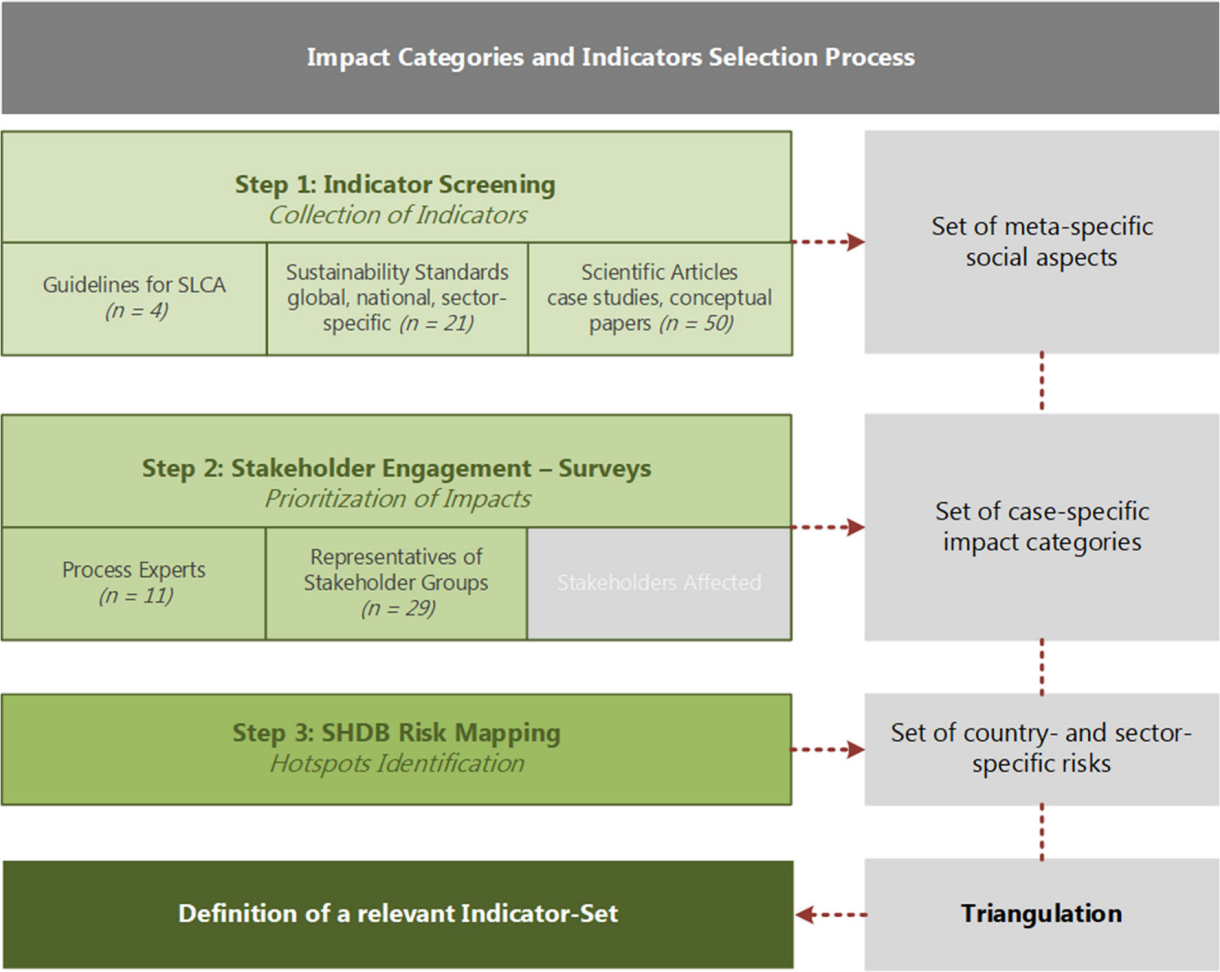
Procedure of indicator selection, fragmentarily adopted from Siebert [19], supplemented with several crucial steps like diversification in stakeholder engagement, risk mapping, and triangulation (own figure)

A multi-methodological approach was chosen to identify and prioritize relevant indicators. The basis of provided indicators is found in the “Methodological Sheets for Subcategories in S-LCA to by Benoît-Norris et al. [40], a supplement to the UNEP/SETAC Guidelines that also offers a vast number of indicators. In accordance with Siebert et al. [19], the selection is based on screening social aspects and impact categories in SLCA guidelines, sustainability standards on a global, national, and sector-specific level and a literature review on articles of SLCA studies in a related context (bio-based value chains). The following subchapters describe the three steps of the indicator-selection approach depicted in Fig. 1. The different steps intend to provide an extensive picture of potential social aspects, going beyond minimal compliance to assess additional and complementary social impacts, as required by Benoît et al. [8]. The combination of the three steps results in an initial hot spotting, including the main social implications occurring along the value chain under investigation.

To the author’s knowledge, there are three common practices for identifying impact categories and indicators in SLCA, summarized in the following three points:

1. Indicator screening: a literature review is carried out to some extent in nearly every study screened so far. The consultation of literature can be seen as the basis for further investigations. Especially a comparable research topic allows to include social aspects from earlier studies [41]. The screening of indicators can include guidelines and instructions for SLCA, sustainability standards on global, national, or sector-specific level [19], and scientific publications in the respective field of interest, including SLCA case studies.
2. Stakeholder engagement: a participatory approach is proposed by several authors (e.g., Benoît et al. [8] or Mathe [29]). Prior to indicator-selection by stakeholders, it is of importance to identify the respective stakeholders.
3. Risk mapping: the consultancy NewEarth B established the Social Hotspots Database (SHDB) and provides purchasable licenses for using the “SHDB Risk Mapping Tool” [42]. This database was chosen because the web-based tool enables the researcher to identify country- and sector-specific hotspots in a time-saving manner. But there are also other databases available for SLCA, such as PSILCA or solca by GreenDelta.

#### Step 1: Indicator Screening (Literature Review)

An indicator screening was carried out reviewing guidelines for SLCA, sustainability standards, and scientific peer-reviewed articles. To the author’s knowledge, four different guidelines are used as the basis for implementing an SLCA, which are (1) the UNEP/SETAC Guidelines for Social Life Cycle Assessment of Products [8], published in 2009; (2) the ISO 14040: International Standard for Environmental management–Life cycle assessment–Principles and Framework [43, 44], published in 2006; (3) the Product Social Impact Assessment (PSIA)–Methodology and Handbook (also referred to as: Pré Sustainability Assessment) [9, 45], published in 2018 as well as (4) the SEEbalance*®* method [46], published in 2004. The consultation of SLCA literature has shown that the UNEP/SETAC Guidelines are frequently referred to in literature. Less attention is paid to the Product Social Impact Assessment (PSIA) and the SEEbalance©, which focus on socio-eco-efficiency analysis. Although ISO 14040 does not offer specific guidance for SLCA, it does provide the basis for LCA and should therefore serve as a basis for SLCA also. Therefore, it is also included in the list of guidelines for SLCA. Information on relevant sustainability standards was gathered by snowballing in reviewed publications and online, mainly using the Google search engine and screening references of related publications. In accordance with Siebert et al. [19] and Bracco et al. [35], the selection of relevant sustainability standards is based on a global, national, and sector-specific level. Obtaining any information about social and socioeconomic impact categories or indicators in sustainability standards at the national level in Slovakia is particularly challenging. However, this work focuses on standards relevant to the bio-based and agricultural sector in general and with a specific focus on Slovakia.

Furthermore, a systematic literature review was carried out. The common search engines Scopus (SCO), Web of Science (WOS), and Cambridge Scientific Abstracts (CSA) were consulted. In accordance with terms used in specific literature, the following different terms for SLCA, like “Social Life Cycle Assessment,” “SLCA,” “S-LCA,” “Social LCA,” and “SOLCA” were used in combination with “Indicator*” and “Impact Categor*” for general results on indicators in SLCA, but also in combination with “Short Rotation Coppice,” “Short Rotation Plantation,” “SRC,” and “SRP” to check for more specific information. Additionally, for SLCA studies on the final bio-based products of concern, terms like “lightweight fibre board,” “eco-fungi,” “moulded fibre,” “WPC,” or “Wood Plastic Composit*” (non-exhaustive listing) were included. Due to the focus on SLCA in this study, no other sustainability assessments were included in the review. This search strategy resulted in 268 (SCO), 630 (CSA), and 193 (WOS) hits respectively for the general studies on impact categories and indicators. The specific search terms on SRC as well as the terms lightweight fibre boards, eco-fungicidal moulded fibre parts, and WPC, which represent the final bio-based products under study, led to a much smaller number of results. Overall, these terms yielded 4 (SCO), 313 (CSA), and 3 (WOS) hits respectively for all terms summarized. In the following, the abstracts of all studies were screened, and the selection of relevant articles was based on addressing the listed thematic areas, implementation of an SLCA in the respective field of interest as well as methodological approaches respective to social aspects and indicators. Grey literature (conference proceedings, dissertations) and studies on a field not comparable (e.g., aquaculture, greenhouse legumes growing, livestock production) were excluded. Finally, 50 studies have been identified as suitable for the content analysis, shown in Table 2, where the reference is assigned to the relevant sector and geographical location of the study. The content analysis was based on the categorization of social impact (sub-) categories, indicators, stakeholders addressed, information on the indicator selection process, and number of indicators stated as well as general information on the scope of the study and the production system. The qualitative data analysis of the publications was done using the software Atlas.ti, to establish an inductive category system. This preliminary work was necessary to aggregate the impacts and indicators collected into relevant social aspects addressed in the different studies. Summarizing the different parts of literature screening from Step 1, a set of indicators on a meta-level was obtained.

**Table 2:** Studies on SLCA providing social aspects and/or indicators for the assessment of agricultural and bio-based production systems as well as conceptual or methodological papers on SLCA indicators.

Nb.	Source	Geographical scope
Type of product/field of application: agriculture		
1	Franze and Ciroth 2011 [47]	Ecuador, Netherlands
2	De Luca et al. 2014 [48]	Italy
3	Vavra et al. 2014 [49]	Czech Republic
4	De Luca et al. 2015 [50]	Italy
5	Tecco et al. 2016 [51]	Italy
6	Arcese et al. 2017 [52]	Italy
7	Sawaengsak and Gheewala 2017 [53]	Thailand
8	Lim and Biswas 2018 [54]	Malaysia
9	De Luca et al. 2018 [55]	Italy
10	Prasara-A and Gheewala 2018 [56]	Thailand
11	Petti et al. 2018 [57]	Italy
12	De Luca et al. 2018 [58]	Italy
13	Prasara-A and Gheewala 2019 [59]	Thailand
14	Iofrida et al. 2019 [60]	Italy
15	Du et al. 2019 [61]	Brazil
16	Du et al. 2019 [62]	Brazil
17	Muhammad et al. 2019 [63]	Malaysia
18	Martucci et al. 2019 [64]	Italy
19	Sawaengsak et al. 2019 [65]*	Thailand
Type of product/field of application: bio-based energy, bio-fuel		
20	Lehmann et al. 2011 [66]	Developing countries
36	Valente et al. 2011 [67]	Norway, USA
21	Macombe et al. 2013 [20]	Finland
22	Henke and Theuvsen 2013 [68]	Germany
23	Manik et al. 2013 [69]	Indonesia
24	Ekener-Petersen et al. 2014 [70]	Brazil, USA, France, Lithuania
25	Weldegiorgis and Franks 2014 [71]	Australia
26	Pashaei Kamali et al. 2014 [72]	Latin America, European Union
27	Dewulf et al. 2015 [73]	-
28	Dos Santos and Brandi 2015 [74]	Argentina, Brazil, China, USA, France
29	Ren et al. 2015 [75]	China
30	Sanchez Ramirez et al. 2016 [76]	Brazil
37	Ekener et al. 2016 [25]	General
31	Contreras-Lisperguer et al. 2018 [77]	Jamaica
32	Sajid and Lynch 2018 [78]	Canada
33	Rafiaani et al. 2018 [33]	General
34	Martin et al. 2018 [16]	Sweden
35	Souza et al. 2018 [79]	Brazil
38	Collotta et al. 2019 [80]	General
Type of product/field of application: bio-based products		
39	Agyekum et al. 2017 [81]	Ghana
40	Falcone and Imbert 2018 [32]	General
41	Spierling et al. 2018 [5]	General
42	Blanc et al. 2019 [82]	General
43	Prasara-A et al. 2019 [18]	Thailand
Type of product/field of application: bioeconomy		
44	Siebert et al. 2018 [19]	Germany
45	Falcone et al. 2019 [14]	General
Type of product/field of application: biorefinery		
46	Cadena et al. 2019 [83]	General
Type of product/field of application: methodological or conceptual papers		
47	Kolotzek et al. 2018 [84]	General
48	Sureau et al. 2018 [85]	General
49	Liu and Aswara 2019 [86]	General
50	Rafiaani et al. 2019 [87]	General

*Belongs equally to the field of application: bio-based energy

#### Step 2: Stakeholder Engagement

To obtain a more comprehensive picture, and to avoid a shift towards specific interests of engaged individuals or groups, a multi-level stakeholder consultation process was implemented. Therefore, subsequent to the literature screening, an online survey was conducted to prioritize the social aspects according to stakeholders’ and experts’ input. A two-step approach was implemented, covering the opinion of process experts, which includes individuals directly involved in the case studies project and representatives of the stakeholder groups involved. The questionnaire was built up based on the work of Karlewski [88], which had the advantage that this survey method has already been tested. Their survey was also carried out to grasp the importance of social aspects for an SLCA in the context of a complex international value chain (automobile). The indirect question method, supplemented with examples should help to better understand the social aspects proposed. For that reasons, this method seemed suitable for our application also. The Stakeholders’ perspective was gauged using a six-point Likert scale ranging from 1 = “very important” to 6 = “not important at all”, to determine the perception on the significance of the social aspects given. This approach is known to weight the sustainability criteria through stakeholders’ preferences, which makes the decision on social indicators more relevant on a local level [50, 89]. A regionally dispersed value chain, as is often found in the bioeconomy, makes it necessary to integrate stakeholders from different regions into the survey [90].

Two surveys are divided, engaging (1) process experts, having detailed knowledge about the production processes going on and (2) stakeholders’ representatives, representing the stakeholder groups concerned. Due to the very specialized group of participants, the survey had to be conducted on a very small scale. An online survey for that kind of stakeholder engagement is a good way to capture the opinions of people from diverse backgrounds. As the target group for the surveys is not a homogeneous group, but rather people with different professional backgrounds, from different countries and language backgrounds, it would have been challenging to involve the stakeholders in the form of face-to-face contact, such as in the course of a workshop. Furthermore, plans to conduct at least parts of the survey within a workshop in the spring of 2020 were disrupted by the COVID-19 pandemic. Two main tasks were given in the course of the survey. On the one hand, the participants were asked to rank the four common stakeholder groups according to their importance to be included in the SLCA. Under an “others” category, participants had the opportunity to name an additional stakeholder group. On the other hand, the participants were asked to rate the priority of including specific social aspects, regarding the stakeholder groups workers (11 aspects), local communities (11 aspects), society (5 aspects), and value chain actors (4 aspects), into the SLCA study. The social aspects to be assessed include all subcategories proposed by the UNEP/SETAC Guidelines. In addition, for the stakeholder group “workers,” the aspects safe working conditions, training and work-life-balance were added, and for the stakeholder group “local community,” the aspects regional value creation and contribution to economic development were added. The aspect respect of indigenous rights was used in a modified form to also include local communities’ rights. With the option “others” the possibility was given to add additional aspects.

The process experts’ survey was carried out prior to the survey of stakeholder’s representatives. This approach allowed an intensive testing of the survey. The process experts selected for the survey are directly involved in the establishment of the production processes or the R&D on the respective value chain and, thus, could be contacted personally. Since they should have an exact picture of which processes are occurring along the value chain, the process experts’ survey was carried out separately. Therefore, in this survey, the opportunity was given to assess the importance of the social aspects in an SLCA for all stakeholder groups. After this survey did not show any problems, and no feedback about poor comprehensibility was received, the survey was distributed to a range of representatives of the stakeholder groups concerned. The target group for the second survey included employee and worker’s representatives (e.g., farmers’ and workers’ associations), mayors of different villages and other representatives of local communities, NGOs on respective issues, as well as business partners, representing value chain actors. A total of around 165 people of interest were found through online research and conversations with process experts. These people were contacted in the spring of 2020 by e-mail with a short explanation of the projects’ goal and were asked to participate on a voluntary basis. After 3 weeks, an e-mail with thanks for the participation and reminding those who had not yet participated was sent out. This approach made it possible to find out the stakeholders’ views and opinions on the social aspects in an anonymous way. Every survey provided a combination of prioritizing given social aspects and adding additional topics for every stakeholder group, as well as adding additional stakeholder groups.

Eleven completed surveys from process experts’, including answers from all addressed organizations, and 29 completed surveys out of 78 in total from the stakeholders’ representatives could be included in the statistics. The respondents were asked which stakeholder group they can represent, where 12 answers were assigned to “workers,” 6 to “local community,” 4 to “society,” 5 to “other value chain actors,” and 9 to “other,” an additional stakeholder group. For the analysis, Microsoft Excel was used to describe the response frequencies. The mean values of the Likert scales were used to obtain the level of agreement on the importance of the social aspects and therefore, a tendency for prioritization.

#### Step 3: Risk Mapping

In the third step, the Social Hotspots Database Risk Mapping Tool was consulted to consider a hotspots mapping on a country- and sector-specific basis. The web-based tool provides the opportunity to include 57 sectors and 191 countries [91]. Suitable for the present study, the sectors “crops” and “wood products” were selected for the country Slovakia. The integrated database is built on international statistics and estimates a potential risk exposure from the level “low,” “medium,” “high” to “very high”. The results were processed in Microsoft Excel.

### Triangulation

After steps 1–3 had been carried out, the results of these parts could be merged by triangulation. The principle of triangulation (originally mentioned by Webb et al. [92]), was applied to improve the reliability [93] and the confidence [94] of the single indicator-sets. A comparison by several applied methods or different data sets allowed us to cross-check the results [94] and highlight different perspectives on the object under investigation [93]. In this context, method-, data-, theory-, and investigator-triangulation were differentiated [93]. In our study, a mix of method- and data-triangulation was applied, which was intended to glean more knowledge about the main social aspects for SLCA. Data triangulation requires the integration of different data sources that should investigate different perspectives in time, places, and people on the same phenomenon [93]. The combination of methods includes “within-method-triangulation” (e.g., different scales in one questionnaire) and “between-method-triangulation” (the use of different methods), to overcome the limitations of one single method [93]. However, the focus should be on a highly critical selection process and on checking the appropriateness of the chosen methods [93]. For our purpose, the results were compared in a table to visualize similarities and contradictions. By complementing the various methods and data sets, the results will be aggregated more densely, deriving higher redundancy, validity, frequency, appropriateness, and reliability.

## Results

The aim of this publication is to identify the most relevant social and socioeconomic aspects and indicators for the SLCA of bio-based value chains, produced from SRC dendromass, especially in Eastern Europe. These so-called “hotspots” are of particular social interest, as these aspects are presumed to have a particularly high potential for conflicts. Therefore, the subsequent presented social and socioeconomic hotspots are of particular relevance to be included into a detailed SLCA. It is recommended to consider these aspects in detail for the respective foreground system in an SLCA study and to assess potential implications in the foreground system as far as possible with primary data.

### Literature Review

#### Guidelines for SLCA:

at the very beginning of the literature review, it makes sense to consult guidelines for the implementation of an SLCA. Four guidelines have been identified that contain a number of social and socioeconomic aspects as well as indicators and provide the methodological framework for carrying out an SLCA. However, it is not possible to conclude on their relevance for any particular application. Table 3 gives insight into the impact categories of the guidelines with their respective stakeholder groups addressed. All guidelines, except the ISO 14040 norm, which does not provide special information about socioeconomic impact categories, but gives the basic methodological framework of life cycle assessment, include the stakeholder groups workers or employees, consumers, users, or customers, as well as local community. The proposed impact categories for workers and employees deal mainly with working conditions (e.g., fair salary, child labor, and discrimination) as well as health and safety issues. Health and safety for consumers, users or customers are also included in all three guidelines. Benoît et al. [8] and Goedkoop et al. [45] propose health and safety impact categories also for the stakeholder group local community, whereas Schmidt et al. [46] focuses mainly on the issues of job provision for this group. Additionally, less common stakeholder groups are proposed by Goedkoop et al. [45], including small-scale entrepreneurs as a stakeholder group of their own. Goedkoop et al. [9] define this group as an independent person, earning their living in family structures by small-scale production of food and non-food products, characterized by limited access to resources. The main issues that may affect this group coincide mostly with the issues of the more general groups workers and local communities. In contrast, Schmidt et al. [46] include the stakeholder groups’ future generations and international community. The social issues concerned with these groups, can be widely associated with the issues concerning the stakeholder group society generally.

**Table 3:**
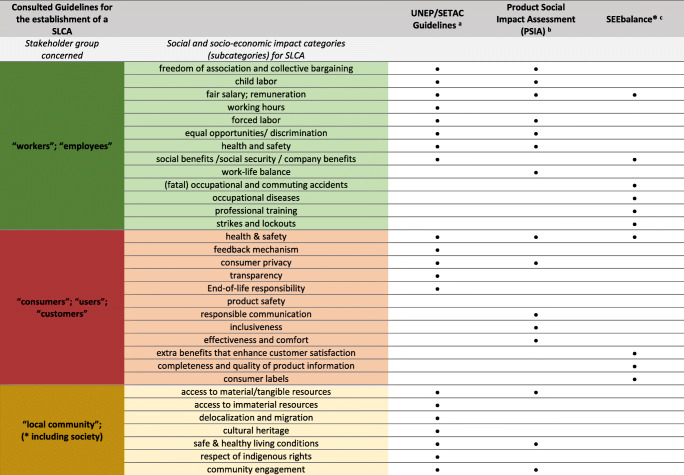

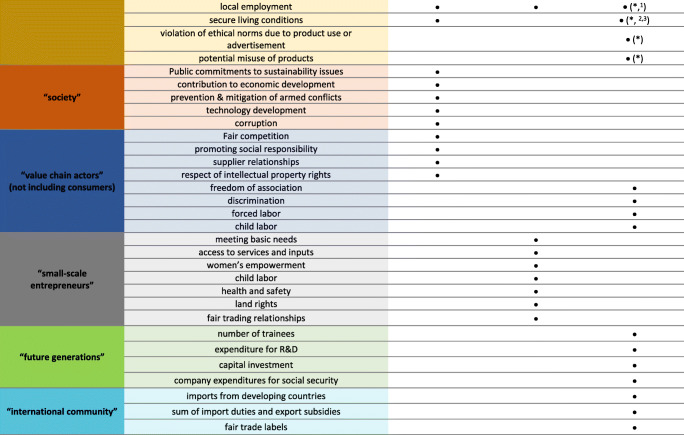
Social and socioeconomic impact categories for SLCA derived from several guidelines

#### Sustainability Standards

A range of social aspects could be drawn from screening sustainability standards on a global, national, and sectoral level. It can be observed that social aspects derived from sustainability standards have a strong focus on workers’ needs (compare Table 4). Almost all standards chosen, except “Greener Slovakia” and the “National Bioeast Hub,” include social aspects that can be assigned to the stakeholder group “workers.” In addition to the stakeholder group workers, social aspects concerning the “local community” or “society” are also frequently addressed. Social aspects regarding “value chain actors” and “consumers” could only be found in GRI standards and the SAFA standard. For the current study, it is especially interesting, that the ILO standards provide an extra convention on plantations (No. 110). Furthermore, the results show that the sector-specific and national sustainability standards lay a particular focus on food security as well as on safety, creation of jobs and rural development. Due to limited space, the table with all social aspects drawn from the sustainability standards is provided in the supplementary material. Table 4 indicates the stakeholder groups that are addressed by the social aspects mentioned in the respective sustainability standard. An estimation of the importance or significance of the respective aspects drawn from the standards is complex. It is challenging to determine the background to the work on which the standards are based and to judge their comprehensiveness. Due to a lack of search criteria, a complete picture of the selected sustainability standards cannot be guaranteed. Global standards are sector and cross-country standards, established by recognized organizations. National standards are based on a general level, as are the global standards. In this study, Slovak standards were sought to address the country-specific social sustainability aspects. The sectoral standards, on the other hand, may again be relevant at the international or EU level. In the present study, sustainability standards for the agricultural sector, and specifically for the bio-based industry, are included. The findings show that many of the social aspects are overlapping, since national or sectoral standards often adopt aspects from global standards.

**Table 4:**
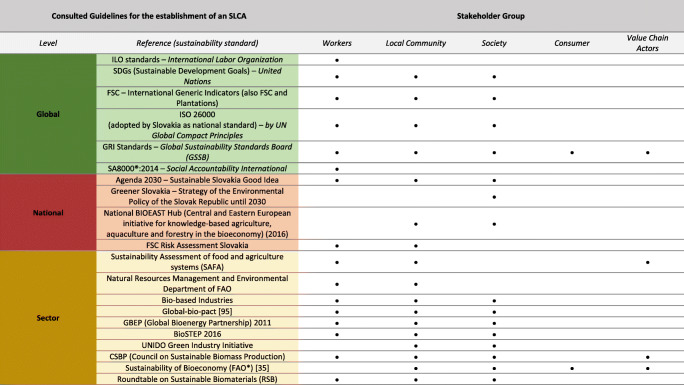
Sustainability standards (global, national, and sector-specific) included in the indicator screening and the stakeholder groups addressed by their social and socioeconomic aspects (the whole list of addressed social and socioeconomic aspects could be found in the supplementary material)

*Food and Agriculture Organization of the United Nations

#### Scientific Publications (Case Studies with Related Focus, Conceptual Papers, etc.)

In our literature review on SLCA, case studies with a related research focus and papers on conceptual SLCA or sustainability indicators have been included. Out of the 50 analyzed studies, only one study is dealing directly with dendromass production in SRC; however, their focus of study was production feeding into energy generation rather than the production of bio-based products. No limits regarding the time of publication were set for the search process. The results showed publications starting in 2011, which highlights that SLCA is still a relatively young field in science. An increasing trend in publications can be identified in 2018, which is in line with publications on SLCA in general (compare Kühnen and Hahn [15] or Spierling et al. [5]). The geographical distribution of the regions addressed shows that, above all countries, of the European Union (18 mentions) with a strong focus on Italy, South-East Asian and South American countries are included in the studies (compare Table 2). Studies for the Eastern European region or Slovakia are missing, except one study for the Czech Republic. This shows that SLCA is still underrepresented in this geographical focus. Most articles found were published in the *International Journal of Life Cycle Assessment* (14 publications), closely followed by the *Journal of Cleaner Production* (13 publications). This is followed by the journal *Sustainability* with just four publications remaining. Interestingly, no article regarding the respective topic was published in the journal *BioEnergy Research*.

**Fig. 2:**
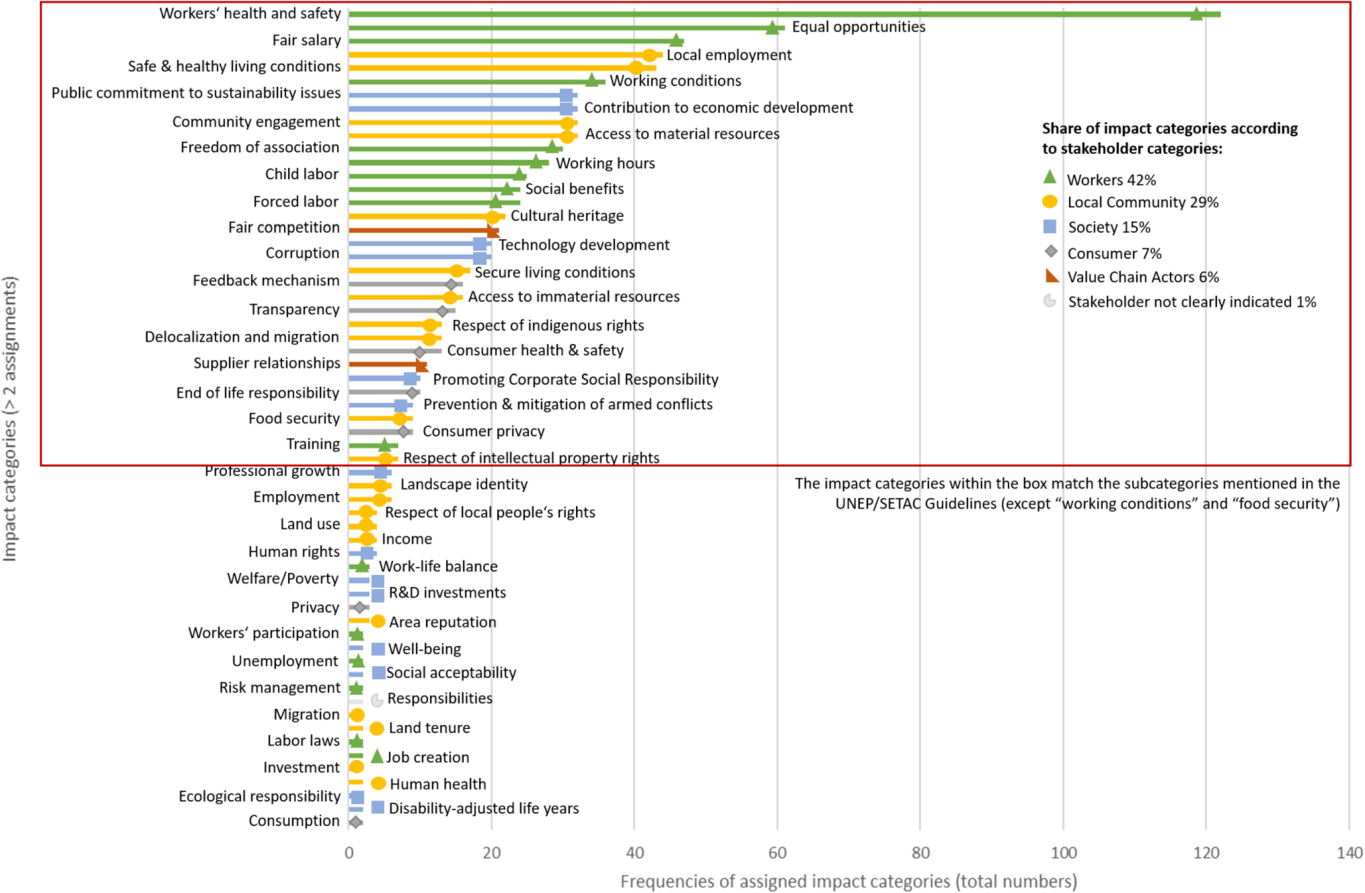
Social and socioeconomic impact categories examined in the scientific publications (*n* = 50)

The analysis of the selected publications focused on the social aspects, more precisely on the social and socioeconomic impact (sub-)categories or the respective indicators. As already discussed in the introduction, the definition of the terms is very controversial, which made the coding of the impact categories and indicators challenging. In order to structure this process, the subcategories of the UNEP/SETAC Guidelines were used as a basis and were supplemented by the impact categories mentioned in the articles. Following this procedure, 118 impact categories were identified. The impact categories addressed show a clear trend towards worker’s health and safety aspects (compare Fig. 2). A strong focus on the stakeholder group “workers” as well as on investigations in the area of health and safety is in line with the findings of other authors. This can be explained simply with respect to the undeniable importance of the topic on the one hand, but it can also be justified by the availability of clearly defined and measurable indicators in this area and should therefore not automatically be seen as the only and most important social aspect in SLCA. It is striking that most of the impact categories assigned refer to the subcategories mentioned in the UNEP/SETAC Guidelines. The 33 most frequently used impact categories completely cover the 31 subcategories of the guidelines; only the impact categories “food security” and “working conditions,” as a more general term for impacts on the stakeholder group “workers,” fall within this range. These impact categories are within the red box in Fig. 2. Additionally, the assignment of the impact categories according to the respective stakeholder group is illustrated in Fig. 2. The reviewed articles show outnumbered impact categories or indicators for the stakeholder group “workers;” 42% of all impact categories are related to this stakeholder group. This reflects the overall situation in SLCA studies, where, in general, a high number of social aspects and indicators is relevant, but a strong focus is placed on workers’ issues [15]. The “local community” is addressed with 29%, “society” with 15%, “consumers” with 7% and “value chain actors” with 6% of all impact categories. This shows that the stakeholder groups “consumers” and “value chain actors” are underrepresented with impact categories in SLCA. Only 1% of the impact categories found could not be clearly assigned to these stakeholder groups.

While it is important to note that the subcategories of the UNEP/SETAC Guidelines were considered as the basis for the coding, it also shows that they provide a rather complete picture of commonly used impact categories or subcategories in their meaning. Nevertheless, this does not mean that additional social aspects are not relevant to the study. Especially, indicators taking the regional or sectoral context into account are of major relevance in SLCA studies [19, 96, 97]. In order to get a grasp of the amount of different indicators or impact categories, their occurrences per article were assessed. This resulted in an average of 19.5 indicators or impact categories per article. While some articles deal with only one single indicator or social aspect, for example, Blanc et al. [82], other articles deal with more than 50 indicators at once (e.g., Sanchez Ramirez and Petti [76] or Martucci et al. [64]). A majority of the studies reviewed conduct stakeholder opinions for the selection of indicators (e.g., Manik et al. [69], De Luca et al. [50], Sawaengsak et al. [65], or Rafiaani et al. [87]). Only a few studies, such as Arcese et al. [52], are built solely on indicators from a literature review or on the impact categories listed in the UNEP/SETAC Guidelines (see also Franze and Ciroth [47] or Lehmann et al. [66]). Only one study has made use of the Social Hotspots Database, which is Ekener-Petersen et al. [70]. They took into account all hotspots tagged with “high” or “very high” risk potential. Stakeholders were involved in the selection or prioritization of indicators in various approaches. Some studies arranged stakeholder workshops to analytical hierarchy processes were installed in the course of surveys, or interviews with individual experts were conducted.

### Prioritized Indicators by the Stakeholder-Survey

**Fig. 3:**
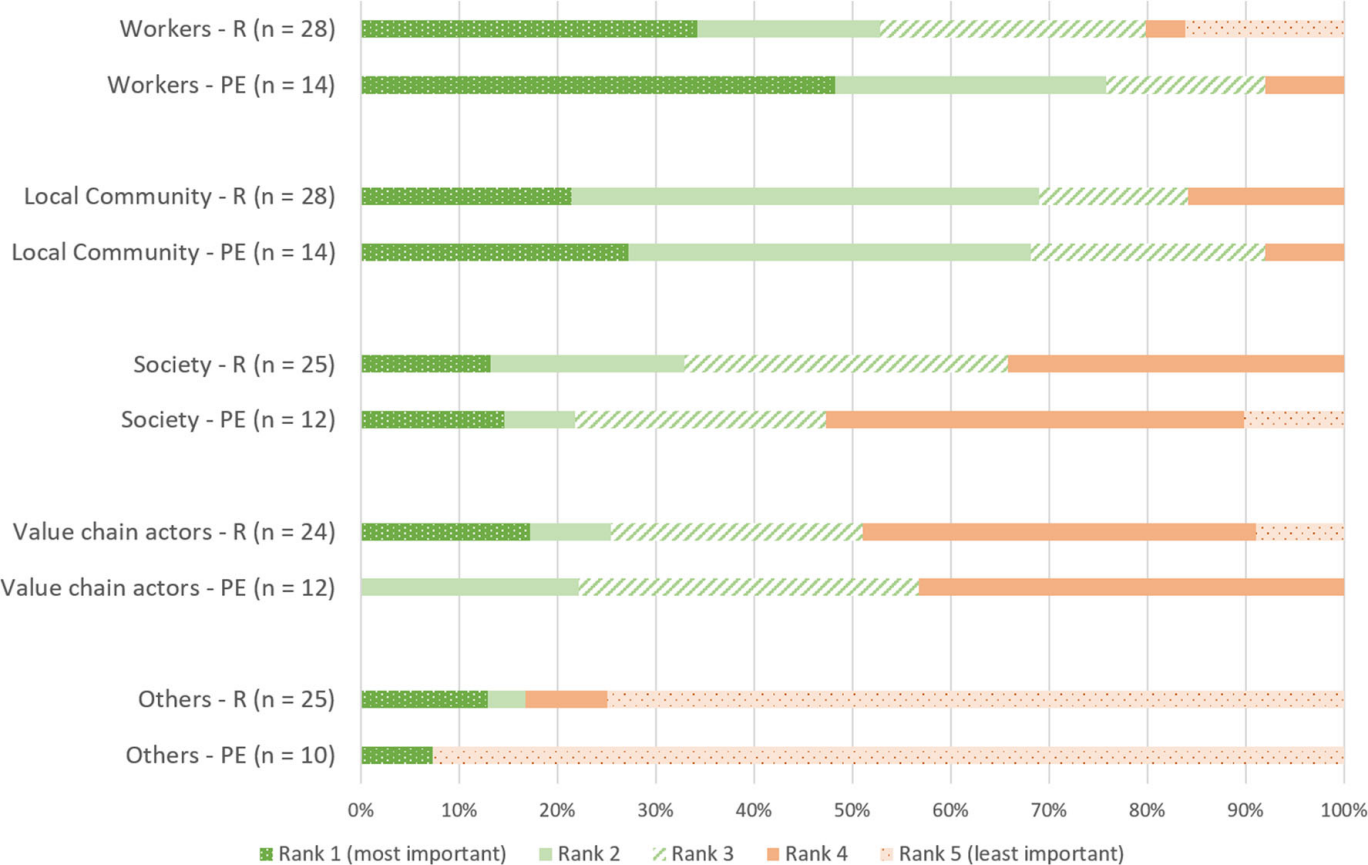
Ranking of the stakeholder groups for prioritization to be included into SLCA (*R r*epresentatives; *PE p*rocess *e*xperts)

In the following section, the results of the stakeholder surveys are presented. The survey participants were divided into two groups; the first group was comprised of “process experts” (PE), having specific knowledge about the value chain under consideration, who had the opportunity to prioritize the impact categories for all stakeholder groups. The second group was formed by various representatives (R) of the different stakeholder groups, who had the opportunity to prioritize the impact categories for the particular stakeholder group they can represent. Figure 3 shows the ranking of the stakeholder groups as prioritized by the survey participants. The results indicate that the survey participants of both groups are in line with the literature review’s results. Nearly 50% of the “process experts” and more than 30% of the “representatives” rank “workers” at the top in terms of importance to include in the SLCA. The second most frequently ranked stakeholder group is the “local community.” Combining ranks 1 and 2, the “local community” is even rated as slightly more important than “workers” by the representatives. The stakeholder categories “society” and “value chain actors” are further down in the list, and also additional stakeholder groups are of minor importance from the participants’ perspective.

**Fig. 4:**
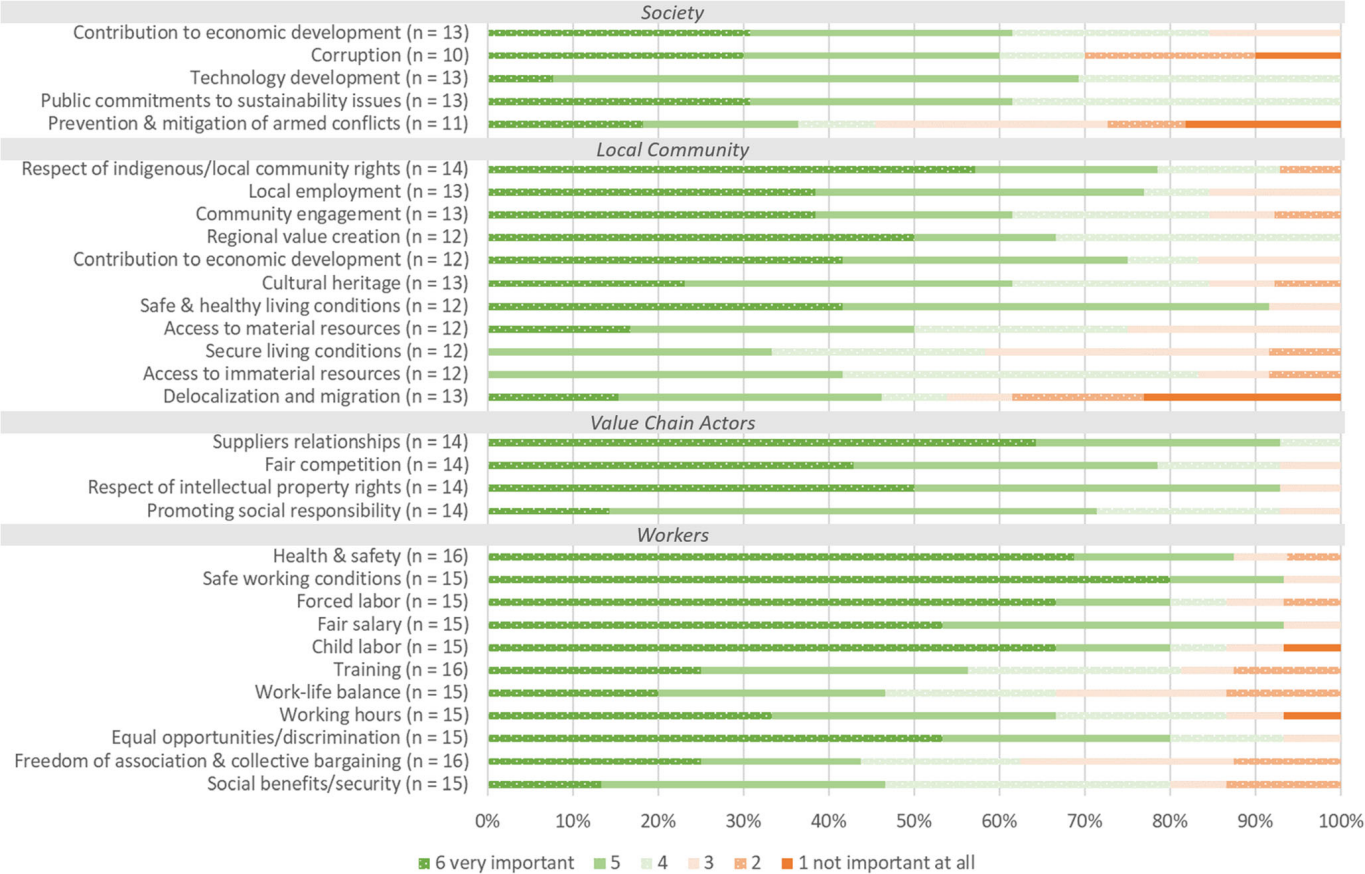
Prioritization of the impact categories by the survey respondents (answers of the two groups “process experts” and “representatives” combined; the differences are shown by the help of mean values and standard deviations in the supplementary material)

Subsequently, the second task in the surveys focused on the social and socioeconomic impact categories. This part of the survey was subdivided regarding the impact categories to the four respective stakeholder groups “workers,” “local community,” “society,” and “value chain actors.” Figure 4 shows the results in frequencies for the prioritization of impact categories to be included in the SLCA. For the simplicity of the graphs, the answers of process experts and representatives have been combined, and the answers given for “others” have been omitted due to low response frequencies. The survey participants were asked for their level of agreement to include the impact category into the SLCA on a six-point Likert scale from “1 not important at all” to “6 very important.” The findings show that the respondents consider safe working conditions, health and safety, forced labor, fair salary as well as working hours and equal opportunities/discrimination as the most important social aspects for the stakeholder group “workers” (compare Fig. 4). Interestingly, the category child labor was ranked many times as “very important” but also received the most rankings as not important at all, especially in the representatives’ survey. This may not indicate that the respondents have no problem with child labor rather than it is not a relevant aspect for Slovakia. In general, less rankings were given for “not important at all” than the rest of the scale. Only the representatives’ survey shows a slight indication that they did not think it is important to include child labor and working hours in the assessment. However, working hours show a completely different picture, when the process experts and the representatives survey are compared. The frequencies for prioritization of the impact categories for the stakeholder group “local community” show that all respondents of the representatives consider the impact categories regional value creation, contribution to economic development, and local employment as “very important.” Furthermore, high priority aspects are also safe and healthy living conditions, access to material resources, respect of indigenous rights/local community, and community engagement. For the group value chain actors, supplier relationships and respect of intellectual property rights are the impact categories ranked as the most important. The impact categories for the group society were generally ranked as slightly less important. However, contribution to economic development was given major priority from the representative’s point of view. Discrepancies in the answers between the groups’ process experts and representatives could be found for the impact category corruption. The process experts considered corruption to be far less relevant to be included into the SLCA than the representatives did. In turn, the process experts found it more important to include working hours in the SLCA than the representatives. Focusing on delocalization and migration in SLCA was more important to the process experts than for the representatives. In order to show the response behavior of the two groups in more detail, mean values and standard deviations provided in the supplementary material are shown for each impact category.

### Risk Mapping

A risk mapping was carried out with the so-called Social Hotspots Database (SHDB) risk mapping tool to gather information about the country- and sector-specific risk potential. A specific sector for SRC or bio-based products industry is not included in the tool. Therefore, the sectors “crops” and “wood products” were considered as relevant for the current study. A total of eight indicators with either “high-” or “very high-” risk potential could be identified for Slovakia. The results are shown in Table 5. The consideration of indicators with “high” and “very high” risk potential was already found in Ekener-Petersen et al. [70]. Most indicators given high priority are covering health- and safety-related issues or evidences for the working conditions.

**Table 5:** SHDB indicators of “high-” and “very high-” risk potential in Slovakia

Indicator	Sector “crops”	Sector “wood products”
Percentage of commercially owned farms	Very high risk	-
CIRI Human Rights Data Project	Very high risk	Very high risk
Malignant neoplasms, estimated age standardized	Very high risk	Very high risk
Average of unemployment percentage at the country level	Very high risk	Very high risk
Workers’ remittances and compensation received	Very high risk	Very high risk
Non-fatal work-related injuries by sector	High risk	High risk
Cardiovascular diseases, estimated age standardized death rate (per 100,000)	High risk	Very high risk
Unemployment percentage at sector level	Low risk	Very high risk

### Triangulation of the Results

By combining the results from the literature screening, from the stakeholders’ surveys and the risk mapping, the most relevant social and socioeconomic impact categories should be determined. Due to the different characteristics, depending on the literature or method consulted, it is necessary to consider them on a consistent level. The results of the different methods applied in steps 1 to 3 are discussed in the following paragraphs.

Table 6 shows the triangulation of these three different steps. Considering all three approaches, strong agreement on the importance to include workers’ and local communities’ aspects into SLCA can be ascertained. The impact categories that were considered to be important by all three methods will be included in the assessment by respective indicators in any case. Also, the impact categories considered to be important by two methods will be included in the assessment. Furthermore, the most important categories chosen by stakeholders’ engagement and risk mapping will be included in the assessment, due to their specific nature. The subsequent Table 7 demonstrates the decision on final indicators, matching the identified impact categories of interest. These indicators make it possible to measure the selected social aspects and impact categories and thus to draw conclusions about their potential positive or negative impacts. Comparing the three steps, it becomes apparent that the impact categories from the indicator screening and the stakeholder engagement are frequently consistent. The risk mapping came up with less social aspects (with high- and very high-risk potential) than the indicator screening and the stakeholder engagement. However, respect of human rights was neither covered by the indicator screening nor by the stakeholder engagement. Through the methods applied, 7 impact categories related to the stakeholder group “worker,” 8 to “local community,” 5 to “society,” and 3 to “value chain actors” should be considered in the SLCA. Considering the described discrepancies in the availability of social impact categories and indicators for the different stakeholder groups, the results fit into this scheme.

**Table 6:**
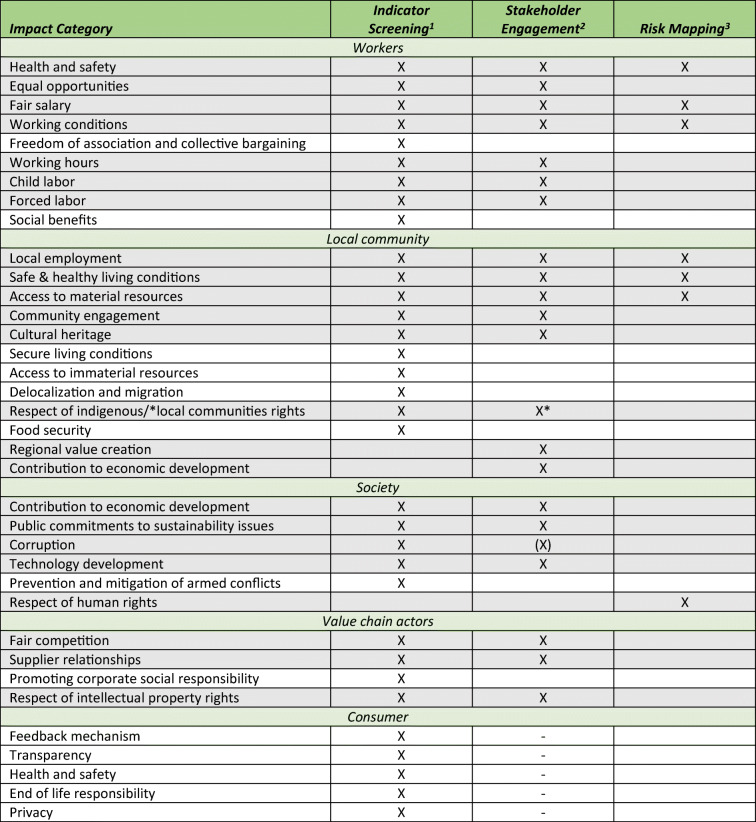
Selection of impact categories based on triangulation of the three different steps (the grey-shaded impact categories will be linked to indicators, to be able to include them in the SLCA)

*X* indicates given high priority; – means not included; ^**1**^Top 33 of the considered impact categories, identified in the indicator screening; ^**2**^Impact categories given highest relevance (= those reaching 50% to be considered as “very important” by either one of the groups (process experts or representatives) or by both counted together; ^**3**^Impact categories referring to the indicators of “high” and “very high” risk potential from the SHDB risk mapping

**Table 7:** Final set of social and socioeconomic indicators for the implementation into the SLCA

Impact categories	Indicators	Units	Measurement description
Workers’ health and safety	1. Occupational accident rate in Slovakia	%	2. Number of (fatal) accidents per year, per employee 3. Number of sick-leave days per year, per employee
	2. Number of occupational (fatal) accidents	nb.	
	3. Sick-leave days per year	nb.	
	4. Exposure to agrochemicals	qual.	
Equal opportunities	1. Country/region gender index ranking	Index	2. Description of potential discrimination practices
	2. Presence of formal policies on equal opportunities	yes/no	
	3. Rate of female workers	%	
	4. Rate of workers from regional minorities	%	
Fair salary	1. Average Slovakian living wage (month)	€	3. Are all employees paid at least by the local basic wage?
	2. Average payment per month, per full-time employee	€	
	3. Payment according to Slovakian living wage	yes/no	
Working conditions	1. Job satisfaction	Index	Job satisfaction index
Working hours	1. Contractual working hours	hours	2. Hours of work per employee/day 3.Hours of consumed holidays per employee/year
	2. Effective working hours (average)	hours	
	3. Effective used holidays	days	
	4. Overtime compensation	qual.	
Child labor	1. Percentage of children working by country and sector	%	1. Description of child labor potential 2. Stating names, birth dates of all workers
	2. Absence of working children under the legal age	yes/no	
Forced labor	1. Evidence of forced labor in the production processes	yes/no	2. Description of working conditions contractually regulated
	2. Workers voluntarily agree upon employment terms	yes/no	
Local employment	1. Unemployment statistics for Slovakia/region	%	
	2. Percentage of workforce hired locally	%	
	3. Number of local full time equivalent created jobs	nb.	
Safe and healthy living conditions	1. Pollution levels by country	%	3. Changes in national/local food prices
	2. Management effort to minimize use of hazardous substances	qual.	
	3. Food security	qual.	
Access to material resources	1. Changes in land ownership	yes/no	
	2. Infrastructure for community access developed	qual.	
Community engagement	1. Number and quality of meetings with community stakeholders	nb./qual.	1. Description of community engagement activities
Cultural heritage	1. Strength of policies in place to protect cultural heritage	yes/no	2. Visual attractiveness and continuity of appreciated landscape heritage [98, 99]
	2. Landscape identity	qual.	
Respect of indigenous/local communities rights	1. Prevalence of racial discrimination	yes/no	2. Description of conflicts, land tenure structures, etc.
	2. Local land rights conflicts/land claims	yes/no	
	3. Annual meetings held with community members	nb.	
Regional value creation	1. Regional value Added	€	
	2. Regional investment, per unit input	€	
	3. Spatial proximity of investments	%/qual.	
Contribution to (regional) economic development*	1. Economic situation of country/region	€/qual.	1. GDP, economic growth, unemployment rates, wage level, etc. 2. Revenues, paid wages, R&D costs, etc.
	2. Contribution to economic progress	€/qual.	
	3. Contribution to household/farm income	€/day	
Public commitments to sustainability standards	1. Existence of public sustainability reporting	yes/no	
	2. Publicly available documents on agreements to sustainability issues	yes/no	
Corruption	1. Risk of corruption in Slovakia/the region	Index	
	2. Commitment to prevent corruption	yes/no	
	3. Anti-corruption program carried out	yes/no	
Technology development	1. Research and development costs spent	€	1. On organizational, sectoral, or project level
	2. Partnerships in R&D	yes/no	
Respect of human rights	1. Slovakian Human Rights Index	Index	1. CIRI Human Rights Data Project
Fair competition	1. Slovakian (sectoral) law and regulations	yes/no	3. e.g., through policies, strategies, formal documents, etc.
	2. Involvement in and performing anti-competitive business practices	yes/no	
	3. Commitment to the prevention of anti-competitive behavior	yes/no	
Supplier relationships	1. Absence of coercive communication with suppliers	yes/no	
	2. On-time payments to suppliers	yes/no	
Respect of intellectual property rights	1. Use of local intellectual property	yes/no	
	2. Policy and practices for compensation of using local intellectual property	qual.	

*Contribution to economic development can be considered either from the perspective of a whole society or from the perspective of a smaller local community

The final set of indicators from Table 7, chosen according to feasibility, is deemed to be relevant for the SLCA of bio-based products with the background of agricultural dendromass production in Eastern Slovakia. However, it must be noted that the adoption of indicators based on a single case study is doubtful [15], due to the specific factors the social impacts are related to.

## Discussion of the Results

Different approaches for the selection of social aspects and indicators have been explored in order to achieve a balanced but most comprehensive picture of relevant social and socioeconomic aspects for the investigated area. The results of the different approaches are discussed in the following paragraphs.

### Guidelines for SLCA

The results show that the social and socioeconomic subcategories from the UNEP/SETAC Guidelines are most frequently referred to in other studies. They are widely used since their introduction in 2009 [34, 100, 101]. This picture can be also obtained by consulting the reviewed literature from Table 4. However, the guidelines cannot guarantee a straight forward implementation, but the included social aspects and indicators can be considered as a basis for the choice of indicators. Especially “the methodological sheets for sub-categories in social life cycle assessment” from Benoît-Norris et al. [40] provide a comprehensive list of social and socioeconomic aspects and indicators. The decision on impact categories and indicators applicable to an entire value chain can be particularly challenging. As already discussed by Karlewski [88], Lehmann et al. [102], or Schebek and Mrani [103], this can be explained by the fact that the focus of the proposed methods lies more on an organizational than on a process level. Variations can be identified in the pre-defined stakeholder groups to be addressed in the study. It cannot be assumed that these stakeholder groups include all persons involved and must be defined more precisely for each case. In this context, the impact categories also need to be assigned to the respective stakeholder group concerned and can vary from the guidelines. ISO 14040 does not provide impact categories or indicators at all; however, an ISO-certified standard for social impact categories and indicators is missing completely. Compared to the UNEP/SETAC Guidelines, the PSIA method does not include “society” or “value chain actors” but includes “small-scale entrepreneurs” as a separate stakeholder group. In contrast, the SEEbalance© method adds “future generations” and “international community” as additional stakeholder groups but also leaves out “society.” Social aspects regarding these additional stakeholder groups being implemented into SLCA studies could not be found by reviewing the scientific publications under consideration.

### Sustainability Standards

These standards can be considered as a practicable source of input regarding the relevance of social and socioeconomic aspects for an SLCA study. This was also recommended by Sureau et al. [85], who differentiated between international treaties, policy documents, and voluntary standards. The division of the standards is in turn similar to the method recommended by Siebert et al. [19], who distinguished between global, national, and sector-specific sustainability standards, which has been adopted for this purpose. Reviewing sustainability standards is not the easiest way to deal with social and socioeconomic aspects, as the results are quite extensive and from this point difficult to structure and to compare. It is questionable whether a screening of all standards is necessary, as our findings show that there are many overlaps, which was also stated by Siebert et al. [19], who identified indicators for an SLCA of a bioeconomy region in Germany. In general, a systematic review on overarching standards, including social and socioeconomic aspects, is challenging and time-consuming. However, especially regional- and sector-specific social aspects of concern can be identified on the basis of sustainability standards. As this study deals with bio-based value chains from SRC dendromass in Slovakia, a focus on regional value creation, on regional economic development, and on food security could be derived from the national and sector-specific sustainability standards. Especially regional value creation was also considered as rather important by stakeholder engagement but was rarely covered in the review of scientific publications. This underlies the importance of a tailor-made SLCA approach for studies related to bioeconomy.

### Scientific Publication

The publications and case studies found show, that the consultation of the UNEP/SETAC Guidelines [8] is common practice. The other guidelines are barely mentioned. It can be assumed, that these guidelines are a helpful source of information, which is useful for the implementation of an SLCA. However, they do not provide any selection or prioritization of impact categories or indicators in terms of thematic (sector) or geographical context differences. Kühnen and Hahn [15] found out that the most frequently addressed impact categories in SLCA are safe and healthy living conditions, promoting social responsibility among value chain actors, consumers’ health and safety, workers’ health and safety, and contribution to economic development. These findings can be confirmed by our study, except that the impact category “consumers’ health and safety” was not one of the most relevant aspects. Also, that workers stood out as the most frequently addressed stakeholder group, followed by local communities and society is consistent with the study of Kühnen and Hahn [15]. By the comparison of different methods, Wu et al. [34] also stated that special emphasis is laid on worker-related aspects, while those related to consumers or value chain actors are less frequently addressed.

### Stakeholder Engagement

We can confirm the findings from Martin et al. [16], stating that the majority of studies consider stakeholder perspectives for the selection on social impact categories and indicators. The main difference between conventional LCA and SLCA lies in the nature of their impact categories. In SLCA, all impact categories describe impacts influencing specific stakeholders. Therefore, the mapping of production processes, which is common practice in LCA, should be accompanied by a stakeholder mapping, to find out the stakeholders of interest within the processes of investigation. As far as the relevant stakeholders are identified, they can be involved in an SLCA study, which gives a voice to potentially affected people and helps to set the focus on the most relevant social aspects of their concern. Furthermore, the involvement of carefully selected stakeholders avoids the implementation of irrelevant indicators into an SLCA study [104]. The findings of our surveys are quite in line with the findings from the literature review. The survey participants prioritized workers’ health and safety aspects and fair working conditions as well as regional value creation and opportunities for the local communities. The study of Falcone et al. [14] focuses also on social aspects for the assessment of bioeconomies and shows a strong focus of stakeholders on workers’ health and safety aspects as well as on human and labor rights, which is in line with our results. Nevertheless, contribution to the economy, which can be seen as parallel to our aspect regional value creation, is not considered as one of the most important aspects. However, it must be taken into account that a non-response bias could not be considered. Additionally, a bias could arise through local surveys, as it is almost impossible to include all stakeholders [89]. The stakeholders’ personal perception can have a strong influence on the results. It is recognized, that participants who benefit from the activities are very active in surveys [105]. Furthermore, a strong focus on local impacts resulted from ignoring the entire life cycle perspective [90] can also lead to a bias.

### Risk Mapping

Also the SHDB risk mapping shows a high relevance of health and safety aspects. These results are close to the study of Mattila et al. [90] who analyzed Finnish wood product supply chains with the SHDB. However, in contrast to the other methods tested, the indicators from the SHDB are not directly linked to specific stakeholder groups. The indicators showing a high-risk potential should raise awareness of hotspots, which should be considered for specific analysis with site-specific data [91, 106]. This allows one to identify actual risks, since the SHDB risk mapping can only point out potential social risks [70].

### The Final Set of Indicators

In the selection of indicators, attention should be paid to choosing measurable, relevant [28, 107] and practical [12, 107] indicators that are sensitive to changes [12] as well as reliable and valid [107]. These selection criteria partly overlap with the benefits of triangulation, thus justifying the adoption of this method. In contrast to conventional LCA, the quantification of impacts can become a real challenge in SLCA [69]. Therefore, qualitative indicators also have an equally important function in the SLCA [106]. As already stated, it is not always appropriate to compare different studies. However, Dewulf et al. [73] suggested indicators for raw materials production, which allow a comparison to some extent. In their study, only worker-related aspects were considered, which match the impact categories health and safety, working hours, child labor, and forced labor from our study. Another relevant study from Dale et al. [12] on socioeconomic aspects for sustainable biofuel production also considered local employment, regional value creation, and contribution to economic development as some of the most important aspects. Additionally, food security was a relevant aspect in their study; this aspect is only covered by the literature review in our study. Malkamäki et al. [108] investigated socioeconomic impacts of global large-scale tree plantations. Their findings underline the importance of an SLCA as the most frequently reported impacts were employment, land, social impacts, and livelihoods. The results indicate that it is justified to adopt a multi-methodological approach to select a balanced set of indicators for an SLCA study. Focusing on a single method bears the risk of under-representing important aspects or laying too much emphasis on less important aspects, as they are highly prevalent in literature for example.

## Conclusions

The identification of relevant social aspects and the selection of appropriate indicators to assess them is the core element of every SLCA. However, little attention is given to the further development of indicator-selection-methods. The study from Siebert et al. [19], concerned with the introduction of a framework for sector- and context-specific indicator selection, was published in 2018. Our study and the findings from literature reveal that there are major differences in the nature of single studies. Differences in the geographical context, also on a micro-regional scale, in the addressing of affected stakeholders and also on sectoral levels need to be considered. Thus, the indicator-sets already established in literature are too unspecific for a direct adoption to our case study. Stakeholder participation, as suggested by several authors (e.g., Benoît et al. [8] or Siebert et al. [19]), should be state-of-the-art and is adopted in several case studies (e.g., Manik et al. [69], De Luca et al. [48], Sawaengsak et al. [65], or Rafiaani et al. [87]). The public opinion on the relevant social aspects to be included in an SLCA, plays a crucial role. Nevertheless, a generalized approach is missing, which is helpful (a) to identify the most important stakeholders, (b) how to choose the most appropriate respondents who represent the stakeholder groups best, and (c) to find a suitable standardized method to reach the respondents and capture their opinion. An answer to these three points would represent a major development of the SLCA method. Due to the specific issues addressed in SLCA, it is likely that this task cannot be solved by a single case study. Although conducting stakeholder involvement with an online survey has the advantage to reach a differentiated target group more easily, it is also challenging to do this without face-to-face contact. Both to explain the intention of the survey to motivate participants and also to address uncertainties, like regarding response rates or to draw conclusions on the intention of the respondents’ choices, become an obstacle. If language barriers discourage from going straight into conversation, online surveys offer the possibility to overcome this barrier. In this context, it can be seen as an advantage to provide written information within the survey. Moreover, the anonymous participation encourages the respondents to answer honestly, without being afraid of any negative consequences due to their answer.

The social topics and indicators frequently discussed in literature and implemented into SLCA case studies are very close to the topics presented in the UNEP/SETAC Guidelines by Benoît et al. [8]. To base the selection of indicators purely on a literature review presupposes that the area under consideration has already been well investigated. However, more and more emphasis is given to topics that have already been applied and are thus presented as increasingly important. This is not necessarily representing the actual situation, since studies are often limited by data availability and thus, important aspects are left out. The SHDB risk mapping tool gives the possibility to get a first overview of the risks quickly. However, it is not guaranteed that the covered sectors within the tool clearly correspond to the observed case, which makes the interpretation of the results challenging. A literature review as well as the use of the risk mapping tool don’t allow for adding new topics to the discussion of SLCA. Even though it is more time consuming and costly, stakeholder engagement becomes crucial for the establishment of new impact categories. To address the concerned stakeholder groups in SLCA with appropriate impact categories and indicators and to involve them in the selection of relevant impacts, require a thorough stakeholder mapping previously.

Due to the limited number of survey participants, the results of our study cannot be generalized for all bio-based value chains in Eastern Europe or even Slovakia. However, an in-depth view was necessary due to the specific conditions, especially differences in the upstream processes of bio-based value chains, and in the geographical to local context. Complex value chains in the bioeconomy make it challenging to represent a complete picture of the affected stakeholders and to reach them directly or their representatives. These limitations lead to additional research topics derived from our study. For investigating relevant social aspects and indicators on a broader scope, a representative study for the Eastern European or Slovakian bioeconomy would be necessary. However, the aforementioned differences must be considered. Nevertheless, a specific look at the selection of indicators based on regional and local characteristics and data availability will be required in any case.

Summarized, the following results can be drawn from the study on hand:

- The results show, that the consideration of the UNEP/SETAC Guidelines has already become state-of-the-art and their subcategories cover the social aspects from the global sustainability standards widely.
- Therefore, special emphasis should be given to national and sector-specific standards.
- The screening of social aspects in scientific publications has shown that a broad range of impact categories and indicators concerning workers’ health and safety and working conditions as well as local communities’ employment and living conditions are available.
- Therefore, stakeholder engagement is recommended in order to not emphasize overrepresented aspects from literature only and to give the opportunity to point out underrepresented aspects.
- However, the findings of our stakeholder engagement show that the survey participants are quite in line with the findings from the literature—they prioritized workers’ health and safety aspects and working conditions as well as aspects related to local communities to be included into an SLCA study. Aspects related to the stakeholder group “society” were less prioritized. Regional value creation and economic development seemed to be important aspects from the point of view of the survey participants—this would not have been evident from literature solely.
- Therefore, it is justified to adopt a multi-methodological approach to select a balanced set of impact categories and related indicators.

The current study shows preliminary results to an SLCA study on bio-based value chains, based on wood from Short Rotation Coppice in Slovakia. The proposed indicator-set serves as a structure for data collection and helps to concentrate on relevant social and socioeconomic aspects, as a starting point for following detailed analysis. A subsequent publication will focus on the application of the indicator-set with a respective case study.

## Supplementary Information

ESM 1 (DOCX 30 kb)

## Data Availability

The datasets generated and analyzed during the current study are not publicly available to guarantee the protection of confidential information but are available from the corresponding author on reasonable request.
