# Supplementary material for: Locating Hotspots for the Social Life Cycle Assessment of Bio-Based Products from Short Rotation Coppice
Source: Bioenergy Res. 2021 Mar 25;14(2):510–33. doi: 10.1007/s12155-021-10261-9 (PMC7993984; doi:10.1007/s12155-021-10261-9)
Supplement: Supplementary file 1 — (DOCX 30 kb) [file 12155_2021_10261_MOESM1_ESM.docx]

**TITLE:**

Locating hotspots for the Social Life Cycle Assessment of bio-based products from Short Rotation Coppice

**AUTHORS:**

*Daniela FÜRTNER^1^, Lea RANACHER^1^, E. Alejandro PERDOMO ECHENIQUE^1^, Peter SCHWARZBAUER^2^, Franziska HESSER (*)^1^*

*(*) Corresponding author
E-Mail address: f.hesser@wood-kplus.at*

**AFFILIATION:**

^1^ Wood K plus – Competence Center for Wood Composites and Wood Chemistry, Kompetenzzentrum Holz GmbH, Altenberger Straße 69, 4040 Linz, Austria

^2^ University of Natural Resources and Life Sciences Vienna, Department of Economics and Social Sciences, Institute of Marketing and Innovation, Feistmantelstraße 4, 1180 Wien

**SUPPLEMENTARY MATERIAL**

Social aspects drawn from screened sustainability standards on global-, national- and sectoral-level (non-exhaustive listing)

| ***Level*** | ***Reference (sustainability standard)*** | ***Stakeholder Category concerned*** | ***Relevant Social and Socio-economic Aspects*** |
| --- | --- | --- | --- |
| **Global** | ILO standards – *International Labor Organisation* | *Workers* | Forced Labor Convention, 1930 (No. 29); Freedom of Association and Protection of the Right to Organize Convention, 1948 (No. 87); Migration for Employment Convention, 1949 (No. 97); Right to Organize and Collective Bargaining Convention, 1949 (No. 98); Equal Remuneration Convention, 1951 (No. 100); Plantations Convention, 1958 (No. 110); Discrimination (Employment and Occupation) Convention, 1958 (No. 111); Minimum Age Convention, 1973 (No. 183); Rural Workers’ Organisations Convention, 1975 (No. 141); Working Environment (Air, Pollution, Noise and Vibration) Convention, 1977 (No. 148); Collective Bargaining Convention, 1981 (No. 154); Occupational Safety and Health Convention, 1981 (No. 155); Occupational Health Services Convention, 1985 (No. 161); Employment Promotion and Protection against Unemployment Convention, 1988 (No. 168); Worst Forms of Child Labor Convention, 1999 (No. 182); Maternity Protection Convention, 2000 (No. 183); Safety and Health in Agriculture Convention, 2001 (No. 184); Violence and Harassment Convention, 2019 (No. 190) |
|  | SDGs (Sustainable Development Goals) – *United Nations* | *Workers*  *Local Community*  *Society* | Goal 1 ‘No Poverty’ (equal rights to economic resources, access to basic services, ownership, control over land, property, inheritance, natural resources, new technology and financial services); Goal 2 ‘Zero Hunger’ (double the agricultural productivity and income of small-scale food producers, ensure secure and equal access to land, other resources and inputs, knowledge, financial services, markets, opportunities for value addition and non-farm employment, implement resilient agricultural practices to increase productivity and production, help maintain ecosystems, strengthen capacity for adaptation to climate change, extreme weather, drought, flooding and other disasters, progressively improve land and soil quality, increase investment, enhanced international cooperation, rural infrastructure, agricultural research and extension services, technology development, plant and livestock gene banks, enhance agricultural productive capacity in developing countries); Goal 3 ‘Good Health and Well-Being’ (reduce number of deaths and illnesses from hazardous chemicals and air, water, and soil pollution and contamination); Goal 5 ‘Gender Equality’ (equal rights to economic resources, access to ownership, control over land and property, financial services, inheritance, natural resources in accordance with national laws, promote women’s empowerment, legislation for the promotion of gender equality and the empowerment of all women and girls at all levels); Goal 8 ‘Decent Work and Economic Growth’ (achieve higher levels of productivity of economies through diversification, technological upgrading and innovation, including through a focus on high value added and labor-intensive sectors, improve progressively global resource efficiency in consumption and production, and endeavour to decouple economic growth from environmental degradation); Goal 10 ‘Reduce Inequalities’ (empower and promote the social, economic and political inclusion of all irrespective of age, sex, disability, race, ethnicity, origin, religion or economic or other status); Goal 11 ‘Sustainable Cities and Communities’ (strengthen efforts to protect and safeguard the world's cultural and natural heritage); Goal 12 ‘Responsible Production and Consumption’ (achieve sustainable management and efficient use of natural resources, promote public procurement practices that are sustainable in accordance with national policies and priorities); Goal 15 ‘Life on Land’ (take urgent and significant action to reduce degradation of natural habitat, halt the loss of biodiversity, protect and prevent the extinction of threatened species); Goal 16 ‘Peace, Justice and Strong Institutions’ (ensure responsive, inclusive, participatory and representative decision-making at all levels) |
|  | FSC – International Generic Indicators (also FSC and Plantations) | *Workers*  *Local Community*  *Society* | Compliance with law (comply with applicable laws, regulations and nationally-ratified international treaties, conventions and agreements); Workers’ rights and employment conditions (maintain or enhance social and economic wellbeing of workers); Indigenous peoples’ rights (identify and uphold indigenous peoples legal and customary rights of ownership, use, management of land, territories and resources); Community relations (contribute to maintaining or enhancing the social and economic wellbeing of local communities); High conservation values (maintain/enhance high conservation values through applying the precautionary approach); Implementation of management activities (management activities consistent with economic, environmental, social policies and objectives) |
|  | ISO 26000  (adopted by Slovakia as national standard) – *by UN Global Compact Principles* | *Workers*  *Local Community*  *Society* | Human Rights (avoidance of complicity, resolving grievances, discrimination and vulnerable groups, economic, social and cultural rights, fundamental principles and rights at work, child labor); Labor practices (conditions of work and social protection, health and safety at work, human development and training at work); Fair operating practices (responsible political involvement, promoting social responsibility in the value chain, anti-Corruption) |
|  | GRI Standards – *Global Sustainability Standards Board (GSSB)* | *Workers*  *Local Community*  *Society*  *Consumer*  *Value Chain Actors* | 401 Employment (employee hires and turnover, benefits, parental leave); 402 Labor/Management Relations (notice periods); 403 Occupational Health and Safety; 404 Training and Education; 405 Diversity and Equal Opportunity; 406 Non-discrimination; 407 Freedom of Association and Collective Bargaining; 408 Child Labor; 409 Forced or Compulsory Labor; 410 Security Practices; 411 Rights of Indigenous Peoples; 412 Human Rights Assessment; 413 Local Communities (engagement, impact assessment, development programs); 414 Supplier Social Assessment; 415 Public Policy (political contributions); 416 Customer Health and Safety; 417 Marketing and Labelling; 418 Customer Privacy; 419 Socioeconomic Compliance; 201 Economic Performance (economic value generated and distributed); 202 Market Presence (entry level wage by gender compared to local minimum wage); 203 Indirect Economic Impacts (e.g. infrastructure investments); 204 Procurement Practices (spending on local suppliers); 205 Anti-corruption; 206 Anti-competitive behaviour |
|  | SA8000®:2014 – *Social Accountability International* | *Workers* | Child Labor; Forced or Compulsory Labor; Health and Safety; Freedom of Association and Right to Collective Bargaining; Discrimination; Disciplinary Practices; Working Hours; Remuneration; Management System |
| **Na-tional** | Agenda 2030 – Sustainable Slovakia Good Idea | *Workers*  *Local Community*  *Society* | Education and Employment; Environmentally sustainable and knowledge-based economy; Sustainability of Settlements, Regions and Countryside in the context of Climate Change; |
|  | Greener Slovakia – Strategy of the Environmental Policy of the Slovak Republic until 2030 | *Society* | Clean Water for Everyone; Protection of Nature and Landscape; Sustainable Land Management; Climate Change and Air Protection; Protection against Flood Consequences; Solutions to Droughts and Water Scarcities; Clean Air; Green Economy; Economic Instruments for a better Environment; Environmental Education and Learning for People of all Ages; |
|  | National BIOEAST Hub (Central and Eastern European initiative for knowledge-based agriculture, aquaculture and forestry in the bioeconomy) (2016) | *Local Community*  *Society* | Strategic thinking in bioeconomy; quality food and feed for Europe and the world; industrial boost for rural areas;  Facing specific societal and economic challenges (climate change and socio-economic characteristics); Coordination of bioeconomy related research and innovation activities; Integrated biomass production for the multi-directional use (management of land, fragmented agrarian structures and marginal areas); Research, analysis of the potential and structural conditions of agriculture as a source of biomass; Strategic directions of development in the bioeconomy; Increasing the value added use of agricultural- and forestry biomass; Motivating knowledge-based modern farming and cooperation among farmers; |
|  | FSC Risk Assessment Slovakia | *Workers*  *Local Community* | Protected sites and species: wood sourced from protection sites (sites with level 3 – 5 of protection according to national legislation, NATURA 2000 areas); Labor rights (specified risk on discrimination of Roma in labor market, specified risk on discrimination of women in labor market; specified risk for gender pay gap); Rights of indigenous and traditional people (specified risk for ILO 169) |
| **Sector** | Sustainability Assessment of food and agriculture systems (SAFA) | *Workers*  *Local Community*  *Value Chain Actors* | Decent Livelihood (quality of life, capacity development, fair access to means of production); Fair Trading Practices (responsible buyers, rights of suppliers); Labor Rights (employment relations, forced labor, child labor, freedom of association and right to bargaining); Equity (non-discrimination, gender equality, support to vulnerable people); Human Safety and Health (workplace safety and health provisions, public health); Cultural Diversity (indigenous knowledge, food sovereignty, |
|  | Natural Resources Management and Environmental Department of FAO | *Workers*  *Local Community* | ESS 6: Involuntary Resettlement and Displacement; ESS 7: Decent Work (creation of more and better employment opportunities, especially for youth and women; non-discrimination and equal opportunity; occupational safety and health; child labor prevention and reduction; forced labor, workers’ and producers’ organizations); ESS 8: Gender Equality (combating discrimination practices; equal opportunities for men and women to participate in and benefit); ESS 9: Indigenous Peoples and Cultural Heritage (identification of indigenous peoples; rights over land, territories and natural resources; prior assessment of the impact on indigenous peoples; free, prior and informed consent; cultural heritage) |
|  | Bio-based Industries | *Workers*  *Local Community*  *Society* | Job creation (especially in rural areas, skilled jobs); Rural Development (involvement of primary producers); Regional Development (diversifying local economy, involving local associations and stakeholders, creating synergies with regional initiatives, mobilising local resources); Competitiveness (of European companies and industry); Science and Knowledge (foster a closer collaboration between scientific community and industry ); Education (society’s awareness about benefits of bio-based products); Standards and Regulations (on the uptake of bio-based products); Safety and Health (developing safer processes and healthier products); Contribution to the UN SDGs (sustainable development) |
|  | Global-bio-pact [95] | *Workers*  *Local Community*  *Society* | Community infrastructure; Community health, welfare, education, etc.; Violation of human rights; Cultural integrity (respect of local culture, traditions, rites); Rights over and access to resources; Cultural heritage; Economic, social and cultural equity; Equal opportunities and discrimination; Economic prosperity; Employment and Income; Secure living conditions; Corruption; Food security; Contribution to local economy; |
|  | GBEP (Global Bioenergy Partnership) 2011 | *Workers*  *Local Community*  *Society* | Price and supply of national food basket; Access to land, water and natural resources; Labor conditions; Rural and social development; Human health and safety; Economic development; |
|  | BioSTEP 2016 | *Workers*  *Local Community*  *Society* | Changing state of health (exposure to agrochemicals, number of multi-resistant organisms, toxicity of “green” vs. “grey” industrial products); access to land, markets, technology (incl. gender issues & tenure); land prices, land tenure, property rights (incl. gender equality); changing employment (new markets) / changing GDP/GNI (change in employment rate, full time equivalent jobs, job quality, need for / lack of highly specialised workforce); changing food security / changing food prices (use of agrochemicals), change in food prices (and its volatility), malnutrition, risk of hunger, macronutrient intake and availability); changing household (income of employees in bioeconomy sector (total), distribution of income); changing nutrition; changing quality of life (change in quality of life, equality); workdays lost due to injury (number of work days lost per worker and year) |
|  | UNIDO Green Industry Initiative | *Local Community*  *Society* | Inclusive and Sustainable Industrialization: commitment to greening of industries; Removal of harmful subsidies; Empowering local authorities and community-based organisations; Education and training to enhance skill base of society; Promoting corporate environmental responsibility; Build up technologies through R&D programmes, technology diffusion, promoting knowledge transfer  Targets of SDG 9 for the promotion of sustainable industrialization |
|  | CSBP (Council on Sustainable Biomass Production) | *Workers*  *Local Community*  *Society*  *Value Chain Actors* | Access to water and other natural resources; Employment, wages and labor conditions; Human health and safety; Good management practices and continuous improvement; Compliance; Participation and transparency; Food utilization; |
|  | Sustainability of Bioeconomy (FAO*) [35] | *Local Community*  *Society*  *Consumer*  *Value Chain Actors* | Support of Food Security (sustainable biomass production, adequate land and natural resources rights, food safety); Ensuring Natural Resources Conservation, Protection and Enhancement; Support Competitive and Inclusive Economic Growth; Make Communities Healthier, more Sustainable and Harness Social and Ecosystem Resilience; Improve Efficiency in the Use of Resources and Biomass; Use of Relevant Knowledge and Proven Sound Technologies, Promote Research and Innovation; Use and Promote Sustainable Trade and Market Practices; Address Societal Needs and Encourage Sustainable Consumption; Promote Cooperation, Collaboration and Sharing between Stakeholders |
|  | Roundtable on Sustainable Biomaterials (RSB) | *Workers*  *Local Community*  *Society* | Human and Labor Rights (no violation of human or labor rights, promotion of decent work, workers’ well-being); Rural and Social Development (contribution to social and economic development of local, rural, indigenous people and communities in regions of poverty); Local Food Security (ensure human right to adequate food, and improved food security); Respect of water-use rights of local people; Use of Technology, Inputs and Management of Waste (minimise risk of dame to environment and people); Land Rights (Respect for traditional land rights of indigenous and local communities) |

*Food and Agriculture Organization of the United Nations

Prioritization of the impact categories by the survey respondents (PE = ”process experts” and R = “Representatives”) shown in mean values and standard-deviations

| ***Impact Category*** | ***Mean Value*** | ***Standard Deviation*** |
| --- | --- | --- |
| ***Workers*** | | |
| Social Benefits / Social Security - PE (n = 9) | 4,2 | 1,1 |
| Social Benefits / Social Security - R (n = 6) | 4,3 | 1,2 |
| Freedom of Association and Collective Bargaining - PE (n = 9) | 4,2 | 1,2 |
| Freedom of Association and Collective Bargaining - R (n = 7) | 4,1 | 1,6 |
| Equal opportunities / Discrimination - PE (n = 9) | 5,6 | 0,7 |
| Equal opportunities / Discrimination - R (n = 6) | 4,8 | 1,1 |
| Working Hours - PE (n = 9) | 5,0 | 0,7 |
| Working Hours - R (n = 6) | 4,3 | 1,9 |
| Work-Life-Balance - PE (n = 9) | 3,9 | 1,0 |
| Work-Life-Balance - R (n = 6) | 4,7 | 1,6 |
| Training - PE (n = 9) | 4,4 | 1,1 |
| Training - R (n = 7) | 4,6 | 1,5 |
| Child Labor - PE (n = 9) | 5,4 | 1,0 |
| Child Labor - R (n = 6) | 4,8 | 1,9 |
| Fair Salary - PE (n = 9) | 5,4 | 0,5 |
| Fair Salary - R (n = 6) | 5,3 | 1,1 |
| Forced Labor - PE (n = 9) | 5,2 | 1,3 |
| Forced Labor - R (n = 6) | 5,3 | 1,1 |
| Safe Working Conditions - PE (n = 9) | 5,8 | 0,4 |
| Safe Working Conditions - R (n = 6) | 5,5 | 1,1 |
| Health & Safety - PE (n = 9) | 5,7 | 0,5 |
| Health & Safety - R (n = 7) | 5,0 | 1,6 |
| Others - R (n = 4) | 4,3 | 1,1 |
| ***Value Chain Actors*** | | |
| Promotig Social Responsibility - PE (n = 9) | 4,6 | 0,8 |
| Promoting Social Responsibility - R (n = 5) | 5,2 | 0,4 |
| Respect of intellectual property rights - PE (n = 9) | 5,6 | 0,5 |
| Respect of intellectual property rights - R (n = 5) | 5,0 | 1,1 |
| Fair Competition - PE (n = 9) | 5,1 | 1,0 |
| Fair Competition - R (n = 5) | 5,2 | 0,7 |
| Suppliers Relationships - PE (n = 9) | 5,6 | 0,7 |
| Suppliers Relationships - R (n = 5) | 5,6 | 0,5 |
| Others - R (n = 2) | 5,5 | 0,5 |
| ***Local Community*** | | |
| Delocalization and Migration - PE (n = 9) | 4,2 | 1,5 |
| Delocalization and Migration - R (n = 4) | 2,0 | 1,7 |
| Access to immaterial resources - PE (n = 9) | 4,0 | 0,9 |
| Access to immaterial resources - R (n = 3) | 4,7 | 0,5 |
| Secure Living Conditions - PE (n = 9) | 3,6 | 1,0 |
| Secure Living Conditions - R (n = 3) | 4,7 | 0,5 |
| Access to material resources - PE (n = 9) | 4,1 | 0,9 |
| Access to material resources - R (n = 3) | 5,3 | 0,9 |
| Safe & Healthy Living Conditions - PE (n = 9) | 5,1 | 0,9 |
| Safe & Healthy Living Conditions - R (n = 3) | 5,7 | 0,5 |
| Cultural Heritage - PE (n = 9) | 4,2 | 1,1 |
| Cultural Heritage - R (n = 4) | 5,5 | 0,5 |
| Contribution to Economic Development - PE (n = 9) | 4,7 | 1,1 |
| Contribution to Economic Development - R (n = 3) | 6,0 | 0,0 |
| Regional Value Creation - PE (n = 9) | 4,9 | 0,9 |
| Regional Value Creation - R (n = 3) | 6,0 | 0,0 |
| Community Engagement - PE (n = 9) | 4,3 | 1,2 |
| Community Engagement - R (n = 4) | 5,8 | 0,4 |
| Local Employment - PE (n = 9) | 4,6 | 1,0 |
| Local Employment - R (n = 4) | 6,0 | 0,0 |
| Respect of indigenous rights/local community - PE (n = 9) | 5,2 | 0,8 |
| Respect of indigenous rights/local community - R (n = 5) | 5,2 | 1,6 |
| Others - R (n = 0) | - | - |
| ***Society*** | | |
| Prevention & Mitigation of Armed Conflicts - PE (n = 7) | 3,1 | 1,8 |
| Prevention & Mitigation of Armed Conflicts - R (n = 4) | 4,3 | 1,3 |
| Public Commitments to Sustainability Issues - PE (n = 9) | 5,0 | 0,8 |
| Public Commitments to Sustainability Issues - R (n = 4) | 4,8 | 0,8 |
| Technology Development - PE (n = 9) | 4,7 | 0,5 |
| Technology Development - R (n = 4) | 5,0 | 0,7 |
| Corruption - PE (n = 6) | 3,5 | 1,9 |
| Corruption - R (n = 4) | 5,3 | 0,8 |
| Contribution to Economic Development - PE (n = 9) | 4,3 | 0,9 |
| Contribution to Economic Development - R (n = 4) | 5,8 | 0,4 |
| Others - R (n = 1) | 1,0 | 0,0 |
